# Evaluation of large-area, low-noise, arrays of SiPMs

**DOI:** 10.1186/2197-7364-2-S1-A6

**Published:** 2015-05-18

**Authors:** Antonio Gonzalez, Albert Talens Aguilar, Hernandez Hernandez, Pablo Conde, Filomeno Sanchez Martinez, José María Benlloch Baviera

**Affiliations:** Institute for Instrumentation in Molecular Imaging, I3M-CSIC, Valencia, Spain

In this work we present the first evaluation results of an array of 12x12 SiPMs MicroFC-30035-SMT from SensL. These sensors are basically identical to those custom fabricated for the brain PET insert of the MindView project, except for the larger package. Both the C-Series and MindView parts are built with the same principle resulting on low dark noise contributions. These arrays cover an active area of approximately 5x5 cm^2^ with a pitch of 4.2 mm. We have especially evaluated the 12x12 MicroFC array with monolithic scintillators. The new results are compared to former findings with the B-Series arrays. In the tests shown here, 10 mm thick black-painted crystals are used, which exit surface dimensions matches the photosensor active area. The photosensor blocks are always run at a stable temperature in the range of 20-22°C (+/- 0.05°C). Typical operation bias of the arrays was 30V, inferring about 600 kHz dark counts rate for each SiPM (67 kcps/mm^2^). A resistive readout has been designed to provide information for each row and column of the SiPMs matrix. Thus, 24 signals are being digitalized, with 250 ns integration time. 9x9 Na-22 collimated sources are used in the experiments. The sources have dimensions of 1 mm in diameter and 1 mm height, and the collimators are 1.2 mm aperture. Spatial resolution results (FWHM) along the crystal surface below 2.5 mm, including source dimensions, have been found. These values are significantly improved compared to 3.5mm obtained with the B-Series in the past. At a distance of 20mm off-center, the system shows a negligible edge effect. These tests also showed a good energy resolution in the range of 16-17%.

